# Noninvasive Methods to Detect Reactive Oxygen Species as a Proxy of Seed Quality

**DOI:** 10.3390/antiox12030626

**Published:** 2023-03-03

**Authors:** Adriano Griffo, Nicola Bosco, Andrea Pagano, Alma Balestrazzi, Anca Macovei

**Affiliations:** 1 Department of Biology and Biotechnology ‘L. Spallanzani’, University of Pavia, Via Ferrata 9, 27100 Pavia, Italy; 2 National Biodiversity Future Center (NBFC), 90133 Palermo, Italy

**Keywords:** DCFH-DA, FOX-1, gene expression, *Glycine max*, heat-shock, ROS, seed priming, seed quality

## Abstract

ROS homeostasis is crucial to maintain radical levels in a dynamic equilibrium within physiological ranges. Therefore, ROS quantification in seeds with different germination performance may represent a useful tool to predict the efficiency of common methods to enhance seed vigor, such as priming treatments, which are still largely empirical. In the present study, ROS levels were investigated in an experimental system composed of hydroprimed and heat-shocked seeds, thus comparing materials with improved or damaged germination potential. A preliminary phenotypic analysis of germination parameters and seedling growth allowed the selection of the best-per-forming priming protocols for species like soybean, tomato, and wheat, having relevant agroeconomic value. ROS levels were quantified by using two noninvasive assays, namely dichloro-dihydro-fluorescein diacetate (DCFH-DA) and ferrous oxidation-xylenol orange (FOX-1). qRT-PCR was used to assess the expression of genes encoding enzymes involved in ROS production (respiratory burst oxidase homolog family, RBOH) and scavenging (catalase, superoxide dismutase, and peroxidases). The correlation analyses between ROS levels and gene expression data suggest a possible use of these indicators as noninvasive approaches to evaluate seed quality. These findings are relevant given the centrality of seed quality for crop production and the potential of seed priming in sustainable agricultural practices.

## 1. Introduction

Seed quality can be defined based on the set of physical, genetic, and physiological characteristics, as per the guidelines given by the International Seed Testing Association (ISTA) [1]. Because seed quality affects germination, its evaluation has become increasingly important for consumers and seed companies, as it constitutes a valuable tool to optimize crop production, with practical and economic benefits [2]. This can be achieved through the development of approaches aimed at determining seed quality in an efficient, noninvasive manner [3]. The current standard approach to monitoring seed viability is mostly based on germination tests which are time-consuming and destructive [4]. Research efforts dedicated to improving seed viability testing are constantly performed, but so far no universal approach has been developed. 

Starting from the primordial state of development on the mother plant, seeds undergo endogenous and exogenous stresses that may undermine cellular structures and functions. As a consequence, reactive oxygen species (ROS) are produced during all phases of seed development, from seed dehydration to storing and germination, posing different outcomes on seed longevity and quality [5,6,7]. In addition to ROS, more recently reactive nitrogen species (RNS) and especially nitric oxide (NO) have also been shown to carry essential functions in seed biology, from their intervention in the regulation of seed dormancy, germination, and aging, to their possible use as seed pretreatments to increase seed quality [8].

ROS production is a side effect of many metabolic pathways (e.g., mitochondrial and plastid electron transport chains, peroxisomal reactions, lipid autooxidation) occurring both under physiological and stress conditions [9,10,11,12]. Uncontrolled ROS accumulation causes oxidative damage and compromises seed viability [13,14]. Aside from detrimental effects, positive physiological functions of ROS were highlighted during the pre-germinative metabolism, related to signaling, dormancy release, reservoir mobilization, and radicle elongation [15,16,17,18]. Thus, ROS play a key role in the activation of pre-germinative metabolism [16,17]. Accumulation of ROS in seeds has been well documented in multiple species and at different developmental stages [19,20,21]. At the cellular level, several components (e.g., mitochondria, peroxisomes, cell membrane, and apoplast) act as preferred production sites. The reactivation of metabolism during seed imbibition causes an enhanced accumulation of ROS, generally resulting from electron leakage within the mitochondrial electron transport chain [22]. Due to their dual nature, ROS must be kept under stringent control by antioxidant defenses. If the balance between ROS production and scavenging is lost, the seeds undergo oxidative stress which can induce seed death. In this view, the presence and diffusion of ROS throughout the cell compartments are spatially and temporally regulated to avoid damage [23,24]. Given the double nature of ROS functions in seeds, the concept of an “oxidative window” of germination is used to evidence this critical range in which ROS can play a positive role in seed metabolism without being detrimental [6].

Fast and uniform seed germination and successful seedling establishment are high priorities for enhancing crop yields. Technologies designed to improve germination performance (generally known as seed priming) can contribute to building up dynamic and sustainable agriculture practices [25,26,27]. Seed priming is the process of regulating seed germination by managing a series of parameters during the initial stages of germination [28,29,30]. For instance, the so-called “on-farm” seed priming, a low-cost technique consisting of soaking seeds in water before sowing, has led to 22% faster seed emergence translated into a 21% yield increase, whereas under stress conditions the plants proved to be more tolerant, gaining up to 22–28% in yield improvements [31]. The main effect of priming is the activation of the metabolic processes triggered during the early phase of germination, or the pre-germinative metabolism [16,27,28]. Although the success of seed priming is strongly correlated to plant species, genotype, seed lot, and vigor, it has also been proven to be effective in improving germination performances during environmental constraints [28,29,32,33,34]. Among the different priming treatments, hydropriming (water soaking with or without aeration) is especially useful in those agricultural areas where crop cultivation is impaired by adverse climate conditions, and it does not require the use of chemical substances [31,35,36,37,38]. Despite its simplicity, hydropriming has been reported to improve germination performances (in terms of germination time, speed, and percentage) in many species [35,36,37,38,39,40,41]. In the case of some practices (e.g., osmopriming, chemopriming), several studies have indicated that these act by delaying water entrance into the seed and thus may limit ROS oxidative injury [42,43,44], whereas in most cases priming acts at the level of seed transition from dormancy toward full germination, touching processes like the activation of DNA repair and antioxidant mechanisms, essential to obtain seeds with improved quality (see comprehensive reviews [16,27,28]). When considering the antioxidant response, enhanced enzymatic activity or increased expression of genes encoding antioxidant enzymes (e.g., SOD, APX, CAT, GR), were evidenced during seed germination and priming treatments [45,46,47,48].

As seed priming is still an empirical procedure, hallmarks useful to monitor the seed response to priming and to discriminate between high- and low-quality lots are necessary to enable the development of efficient protocols [5,35,36,37]. Because ROS play a vital role in seed dormancy and germination, measuring their levels can provide relevant information about seed viability under different conditions. ROS levels have been evaluated as a possible indicator of overpriming seed performance in *Medicago truncatula*, showing that their accumulation during dehydration positively correlates with the loss of desiccation tolerance [49]. ROS levels were also used as a tool to monitor seed quality in *Solanum melongena*, pinpointing that low-quality seed lots defined by low germination rates were characterized by enhanced accumulation of ROS [35]. Additionally, in *Pisum sativum,* accessions with low ROS levels were associated with long-lived seeds, which maintained good germination profiles, whereas short-lived seeds were characterized by higher ROS accumulation [50].

Despite recent advances, the thresholds at which ROS induces toxicity are unknown and conditioned by many factors. Moreover, the necessity to avail of a palette of universal, cost-effective, and noninvasive tools or techniques to monitor seed quality, is highly requested by seed technologists working in industry and seed banks. To this purpose, the current study employed two different biochemical assays, namely dichloro-dihydro-fluorescein diacetate (DCFH-DA) and ferrous oxidation-xylenol orange (FOX-1), to quantify ROS levels in seeds subjected to contrastive treatments leading to enhanced (hydropriming) or impaired (heat-shock) seed quality, in different plant species. These data were also integrated with the expression profiles of genes encoding enzymes involved in ROS production and scavenging.

## 2. Materials and Methods

### 2.1. Seed Materials and Treatments

Seeds of *Glycine max* (commercial variety, provided by Sipcam Oxon SpA, Milan, Italy), *Solanum lycopersicum* (commercial variety, provided by ISI Sementi S.p.A., Fidenza, Italy), and *Triticum aestivum* (commercial variety, provided by ITQB NOVA, Oeiras, Portugal) were used. The seeds were collected from the respective providers and stored at 4 °C until use.

**Figure 1:**
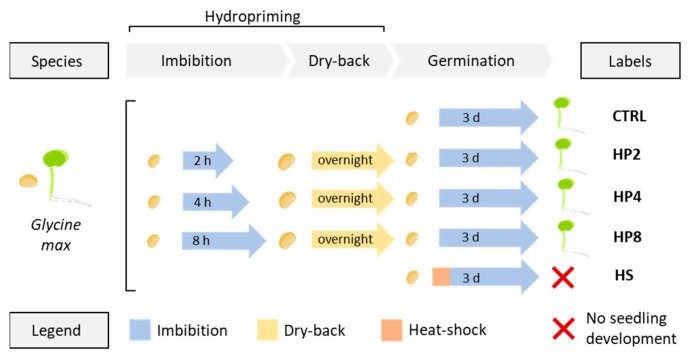
Example of the experimental system applied to *Glycine max* seeds. Imbibition steps are indicated in blue, dry-back is indicated in yellow, and heat-shock is indicated in orange. CTRL, non-treated control; HP2, hydropriming for 2 h; HP4, hydropriming for 4 h; HP8, hydro-priming for 8 h; HS, heat-shock.

Hydroprimng (HP) treatments were conducted in a species-specific manner, especially regarding the time spent during the seed imbibition phase. For instance, in soybean seeds, HP treatments were carried out for 2, 4, and 8 h of imbibition (Figure 1). Considering the *S. lycopersicum* and *T. aestivum* systems, the HP treatments based on seed imbibition in water were carried out at different intervals of time, namely 2 h, 8 h, 24 h for tomato, and 2 h, 4 h, 6 h for wheat seeds. Subsequently, the seeds were air-dried overnight at room temperature to perform the dry-back (DB) phase of the priming treatment. The heat-shock (HS) treatment was carried out in an oven at 90 °C for 30 min for *G. max* and *T. aestivum*, while *S. lycopersicum* seeds were kept in the oven for 3 h. Nontreated controls (CTRL) consisting of dry seeds were also used in the experimental system. A schematic representation of the experimental design representative for *G. max* treatments is given in Figure 1.

### 2.2. Germination Parameters

For germination tests, treated/untreated seeds were monitored in parallel compatibly with the guidelines provided by ISTA [1]. For this purpose, all germination tests were performed in triplicate, where 20 seeds/replicate were placed in Petri dishes containing a filter of blotting paper moistened with 2.5 mL water. All containers were kept in a growth chamber at 25 °C under light conditions with a photon flux density of 150 μmol m^−2^ s^−1^, a photoperiod of 16/8 h, and 70–80% relative humidity. Germination was assessed daily for a period of three days for soybean, five days for wheat, and six days for tomato seeds.

At the end of the indicated periods, the following germination indices were calculated: germinability (G), peak value (PV), mean germination time (MGT), mean germination rate (MGR), coefficient of velocity (CVG), uncertainty index (U), and synchronicity index (Z) [51]. G is defined as the percentage (%) of germinated seeds at the end of the germination test. PV is the highest ratio between the number of germinated seeds at a given time point and the corresponding time, providing an indication of germination rates both in terms of percentage and speed. MGT calculates the average germination time, in which lower MGT values indicate a faster germination. MGR is calculated as the reciprocal of MGT, and it provides an estimation of germination frequency, in which higher MGT values correspond to higher germination frequency. CVG is calculated as the MGR expressed in percentage (%); hence it provides an estimation of germination frequency, in which higher CVG values correspond to higher germination frequency. U is associated with the distribution of germination during the germination test timespan, and higher U values indicate lower synchronization and more dispersion. Z is relative to the synchrony of germination during the experimental monitoring, in which higher Z values indicate high degree of synchronization and lower dispersion in time [51]. The formulas used for the calculation of these parameters is given in the Supplementary Table S1.

Aside from the aforementioned germination parameters, seedling growth was biometrically assessed. The seedling growth was monitored at the end of the experiment by using ImageJ (https://imagej.nih.gov/ij/) software. To this purpose, at least five seedlings/replicate were photographed and used to determine the seedling length (in terms of roots and/or aerial parts).

### 2.3. DCFH-DA Assay

ROS levels were quantified in CTRL and HP, HS, and DB seeds. The assay was carried out by using the fluorogenic dye 2,7-dichlorofluorescin diacetate (DCFH-DA; Sigma-Aldrich, Milan, Italy). The dye is converted to a nonfluorescent molecule following deacetylation mediated by esterases, and it is subsequently oxidized by ROS into the fluorescent compound 2,7-dichlorofluorescein (DCF), which can be detected by fluorescence spectroscopy with maximum excitation and emission spectra of 495 nm and 529 nm, respectively. The assay was carried out as described by Pagano et al. [49], with the following modifications. Seed samples were incubated for 30 min with 500 μL of 10 μM DCFH-DA (for *G. max* and *T. aestivum*) and 70 μL of 50 μM DCFH-DA (for *S. lycopersicum*) or for 15 min. Subsequently, the fluorescence sensor (at 517 nm) of the Rotor-Gene 6000 PCR apparatus (Corbett Robotics, Brisbane, Australia) was used, setting the program for one cycle of 30 s at 25 °C. A sample containing only DCFH-DA was used as a negative technical control to subtract the baseline fluorescence. Data were expressed as relative fluorescence units (RFU).

### 2.4. FOX-1 Assay

Peroxyl radicals and hydrogen peroxide (H_2_O_2_) concentrations were quantified in control and treated seeds at the indicated time points as presented in Section 2.1. The assay was carried out by using the reagent xylenol orange (Carlo Erba, Milan, Italy), which reacts with Fe^3+^ (derived from the oxidation of Fe^2+^ induced by peroxyl radicals and H_2_O_2_) to give a blue-violet complex with an absorption maximum at 560 nm. The working solution (FOX-1 solution) was prepared as described by Bridi et al. [52]. A solution containing ammonium ferrous (II) sulphate (NH_4_)2Fe(SO_4_)2·6H_2_O 25 mM (Merk’s Reagents, Darmstadt, Germany) in H₂SO₄ 0.25 M (Honeywell, Charlotte, NC, USA) was added to a Milli-Q water solution containing Xylenol Orange 125 µM (Carlo Erba, Milan, Italy) and D-sorbitol 100 mM (Duchefa Biochemie, Haarlem, The Netherlands) in a ratio of 1:100. The solutions were mixed gently until the color became uniform. Seed samples were incubated in a sufficient volume (1.5 mL for *S. lycopersicum* and 3 mL for *T. aestivum* and *G. max*) of FOX-1 working solution to allow seeds complete immersion for 45 min. Five replicates of one seed each were used per sample. Subsequently, 1 mL of medium was recovered from each sample and the absorbance was determined at 560 nm by using a Biochrom WPA Biowave (Biochrom Ltd., Cambridge, United Kingdom) spectrophotometer. A calibration curve (Figure S1) was performed by using FOX-1 solution with different concentrations (0, 1.25, 2.50, 5 µM) of H_2_O_2_, and data were represented as [ROOH] concentration values.

### 2.5. Quantitative Real-Time-Polymerase Chain Reaction (qRT-PCR)

Total RNA was isolated from *G. max* treated/untreated seeds by using TRIzol^TM^ (Thermo Fisher Scientific, Monza, Italia), as indicated by the provider. Subsequently, a DNase (Thermo Fisher Scientific) treatment was performed. RNA was quantified by using NanoDrop (Biowave DNA, WPA, Thermo Fisher Scientific). Subsequently, cDNAs were obtained by using the RevertAid First Strand cDNA Synthesis Kit (Thermo Fisher Scientific) according to the manufacturer’s suggestions.

The qRT-PCR reactions were performed with the Maxima SYBR Green qPCR Master Mix (Thermo Fisher Scientific) according to the supplier’s indications, by using a CFX Duet, Real-Time PCR System (BIO-RAD, Milano, Italy). Amplification conditions were as follows: denaturation at 95 °C for 10 min, and 45 cycles of 95 °C for 15 s and 60 °C for 60 s. Oligonucleotide sequences (Table S2) were designed by using Primer3Plus1 (https://primer3plus.com/) and verified with Oligo Analyzer.2 (https://eu.idtdna.com/pages/tools/oligoanalyzer). Relative quantification was carried out by using the *CYP* (peptidyl-prolyl cis-trans isomerase) and RP40S (ribosomal protein 40S) as reference genes [53]. Raw fluorescence data provided by Software 1.7 (BIO-RAD) were used to determine the threshold cycle number (Ct) values for each transcript quantification. Relative quantification of transcript accumulation was performed as described by Thomsen et al. [54] by using the *X*_0_ method in which a conversion of the exponentially related Ct values is introduced to arrive to linearly related values, representing the amount of starting material in a qPCR reaction. All reactions were performed in triplicate.

The choice of investigated genes was based on in silico gene expression data mining obtained from *Arabidopsis thaliana* and *G. max* orthologues, recovered from BAR ePLANT (http://bar.utoronto.ca/eplant_soybean/) [55] and Arabidopsis eFP browser (http://bar.utoronto.ca/efp/cgi-bin/efpWeb.cgi) [56], respectively. The selected genes encoding enzymes involved in ROS production and scavenging include superoxide dismutase (*SOD*1, Phytozome accession No. Glyma.19G240400) manganese superoxide dismutase (*MnSOD*, Phytozome accession No. Glyma.04G221300), catalase 1 (*CAT*1, Phytozome accession No. Glyma.06G017900), catalase 5 (*CAT*5, Phytozome accession No. Glyma.17G261700), ascorbate peroxidase 2 (*APX*2, Phytozome accession No. Glyma.12G073100), respiratory burst oxidase homolog E2 (*RbohE*2, Phytozome accession No. Glyma.08G005900), and respiratory burst oxidase homolog C2 (*RbohC*2, Phytozome accession No. Glyma.06G162300).

### 2.6. Statistical Analyses

Germination data were analyzed with the Student *t*-test by using the Microsoft Excel package using as threshold the *p*-value < 0.05 (‘*’). Estimation of oxidative stress and ROS data were analyzed by two-way ANOVA and Tuckey–Kramer test, where *p* < 0.05 is considered as significantly different, by using the software developed by Assaad et al. [57]. Letters were used to indicate significant differences among all samples. For correlation analyses, Pearson’s correlation coefficient and the relative *p*-values were determined by using MetaboAnalyst 5.0 (https://www.metaboanalyst.ca/) [58]. The same software was also used for principal component analysis (PCA) performed by using the germination parameters and ROS detection data. The obtained “biplot” and “score plot” graphics show how the different sample groups are clustered according to the results obtained in the performed analyses.

## 3. Results

### 3.1. Hydropriming Improves Germination Performance in Multiple Species

**Figure 2:**
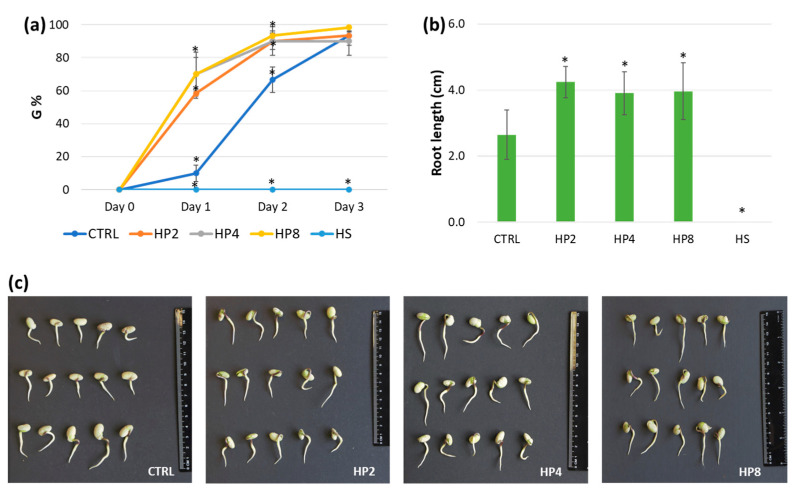
Evaluation of hydropriming efficiency in *Glycine max* seeds. (**a**) Germination percentage (%). (**b**) Root length (cm). (**c**) Representative images of germinated soybean seedlings after three days of treatments. Statistical differences among treatments and control are represented with asterisks (*). *p* < 0.05. CTRL, non-treated control; HP2, hydropriming for 2 h; HP4, hydropriming for 4 h; HP8, hydropriming for 8 h; HS, heat-shock.

Because hydropriming has been already proven to be effective in improving seed germination potential [31,35,36,37,38], this initial part of the work was dedicated to select the most appropriate time points of seed imbibition as this is generally dependent not only on plant variety/genotype but also on the respective seed lots [28,36]. To test the efficiency of hydropriming treatment in improving seed germination, we first focused on soybean, as it is one of the most cultivated species that dominate global agriculture [59]. It has a sequenced and well-annotated genome [60], with data present in several bioinformatics platforms (e.g., Phytozome, BAR ePLANT). Due to the time-specific sensitivity of hydropriming treatments, several imbibition time points were tested. Additionally, to develop a competent experimental design, HS treatments were implemented to decrease seed quality and germination were included along with the control (CTRL). The subsequent analyses indicated that hydropriming resulted in a significantly enhanced germination percentage (G%) compared to CTRL (Figure 2a) for all the tested treatments during the first two days of monitoring. This improved G% was also translated into significantly enhanced root growth when measured at the end of the experiment (after three days) (Figure 2b,c). Data collected for other germination parameters (Table 1) confirm that HP improves germination performance. Specifically for soybean, all the imposed HP treatments showed significant differences compared to CTRL, in terms of PV, MGT, MGR, and CVG, whereas the U and Z parameters were improved only with the HP4 treatment. This indicates that the best time point at which to stop the priming treatment for this soybean commercial variety is after 4 h of imbibition in water, a treatment that brings positive outcomes in terms of germination percentage, speed, and uniformity. The HS treatment was highly damaging and no seed germinated, showing that this can be used as a system to decrease seed quality.

To confirm that these treatments can be universally implemented for different plant species and seed types, additional experiments were carried out by using *S. lycopersicum* and *T. aestivum* seeds (Figures S2 and S3, Table S3). The gathered results show that HP2 and HP8 treatments were efficient in improving germination and seedling growth in tomatoes, whereas for wheat the only significant data regarded G% after one day of monitoring. The results indicate that hydropriming treatments are efficient in boosting germination, but the imbibition time points need to be tailored for each species/genotype/seed lots, as evidenced in other cases [35,36,37,38].

### 3.2. ROS Profiles Are Influenced by the Applied Treatments

Once we have shown that the implemented experimental system can be used to boost or damage seed quality (in terms of germination performance), the next set of analyses was dedicated the evaluate different protocols to measure ROS levels. The two assays hereby employed, namely DCFH-DA and FOX-1, have different targets and specificities. Quantification based on the use of the DCFH-DA molecule measures the general oxidative status [61]. In particular, in the absence of metal or enzymatic catalysts, the DCFH_2_ molecule (produced in the cells through the activity of esterases) is not able to react with some ROS (e.g., O_2_^·−^, LOO^−^, H_2_O_2_) whereas it can react with oxygen and nitrogen radicals, including ·OH and peroxynitrites [61]. In contrast, the FOX-1 assay directly detects peroxidic radicals (ROO˙) and in particular H_2_O_2_ released in the medium whereas it has a low reactivity towards other molecules [62,63].

**Figure 3:**
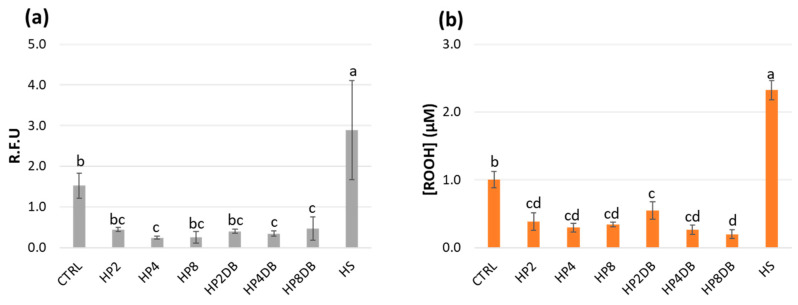
ROS detection in *G. max* seeds subjected to hydropriming and HS treatments. (**a**) Data collected by using the DCFH-DA fluorimetry assay and represented as relative fluorescence units (RFU). (**b**) Data collected from the FOX-1 assay through spectrophotometric measurements and represented as [ROOH] concentration values. Statistically significant differences (*p* < 0.05) are indicated by the occurrence of different letters. CTRL, non-treated control; HP2, hydropriming imbibition for 2 h; HP4, hydropriming imbibition for 4 h; HP8, hydropriming imbibition for 8 h; HP-DB, dry-back treatment following hydropriming imbibition; HS, heat-shock.

Both assays were used to assess these different aspects of ROS accumulation or release by using whole seeds. The gathered data show that the highest amount of oxidative stress (Figure 3a) and peroxide radicals (Figure 3b) are registered in seeds treated with HS. Moreover, the seeds subjected to HP treatments appeared to have the lowest levels of measured ROS, using both the DCFH-DA and FOX-1 assays (Figure 3a,b).

To validate these results, the two assays were applied to the tomato and wheat seeds following the same experimental approaches (Figure S4). Although the FOX-1 results maintained a similar pattern of ROS being released from the seeds (Figure S4b,d), the DCFH-DA assay presented much more elevated levels of variability (Figure S4a,c).

**Figure 4:**
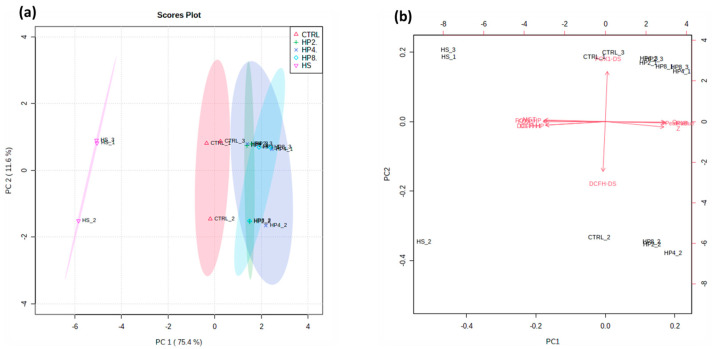
PCA using data gathered for the imposed treatments (CTRL, HP2, HP4, HP8, HS) for *G. max*. (**a**) Score plot grouping of samples subjected to different treatments. (**b**) Biplot obtained with data from germination tests (G, PV, MGT, Z, Rad) and ROS measurements (FOX-1, DCHF-DA) on the clustering of the groups subjected to the different treatments. Because the data provided consisted in triplicate values, the designation _1, _2, _3 in the plots refers to the replicate number.

Subsequently, a PCA analysis was carried out to evidence how the different treatments (CTRL, HP, and HS) are clustered according to the obtained data. The clustering of the group of seeds subjected to the imposed treatments in *G. max* (Figure 4a) shows a distinct grouping of the HP treatments compared to CTRL and HS.

According to the biplot generated by the PCA analysis (Figure 4b), the main contributors to this this clustering are the germination parameters. The CTRL samples clustered separately from the groups subjected to HP and HS treatments, mainly due to the data obtained from the FOX-1 and DCHF-DA analyzes. Finally, the separation of the HS group is, according to the PCA analysis, mainly due to the values obtained for MGT as well as FOX1 and DCHF-DA (Figure 4b). Similar patterns of clusters were also obtained for tomato and wheat (Figure S5).

### 3.3. ROS-Related Gene Expression Is Induced by Hydropriming Treatments

**Figure 5:**
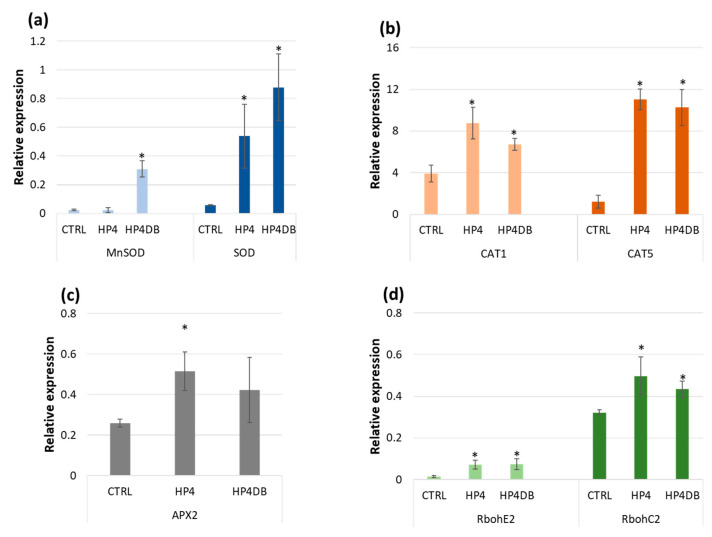
Relative expression of genes encoding enzymes involved in ROS scavenging and production mechanisms in *G. max* seeds subjected to hydropriming treatments. (**a**) Superoxide dismutases, *MnSOD* and *SOD*1. (**b**) Catalases, *CAT*1 and *CAT*5. (**c**) Ascorbate peroxidase *APX*2. (**d**) Respiratory burst oxidase homologs, *RbohE*2 and *RbohC*2. Statistically significant differences obtained by using the Student *t*-test (*p* < 0.05) are indicated with an asterisk (*). CTRL, untreated seeds; HP4, seeds soaked for four hours in water; HP4DB, seeds soaked for four hours and subjected to the desiccation required by hydropriming protocols.

To better investigate ROS homeostasis in the proposed working system, qRT-PCR analyses were carried out to quantify the relative expression of genes encoding enzymes involved in ROS scavenging and production. To select which genes would provide the most relevant information, a preliminary *in silico* data mining approach was conducted simultaneously for *A. thaliana* and *G. max* models. Data relative to multiple isoforms of *CAT, SOD, APX*, and *Rboh* genes were comparatively examined during seed maturation in soybean as well as during the early phases of seed germination in Arabidopsis (Figure S6). This analysis showed that different isoforms of the studied genes are differently expressed during soybean seed maturation with the highest expression being most prevalent for 14DAF (days after flowering), 21DAF, and 35DAF, whereas for Arabidopsis the majority of genes are highly expressed at 48 h of seed imbibition/germination. Based on this investigation, the following genes were selected to perform the qRT-PCR analyses during soybean HP treatment: *MnSOD, SOD1, CAT1, CAT5, APX2* as genes encoding enzymes involved in ROS scavenging, and *RbohE2, RbohC2* as genes encoding enzymes involved in ROS production. Their expression levels were evaluated in soybean dry seeds (CTRL), in seeds subjected to 4 h imbibition (HP4) as the most promising HP timepoint to boost germination, as well as after dry-back (HP4DB) as the last phase of the HP treatment (Figure 5). The gathered data revealed that all genes were significantly upregulated both after seed imbibition (except for *MnSOD*) as well as after the dry-back treatments (except for *APX2*), as compared with the CTRL samples. Interestingly, this trend was common for genes encoding enzymes involved in ROS scavenging as well as ROS production mechanisms. Nonetheless, the highest gene expression levels were registered for the two CAT genes (ROS scavenging) whereas the lowest expression was observed for the *Rboh* genes (ROS production), namely *RbohE2* gene.

**Figure 6:**
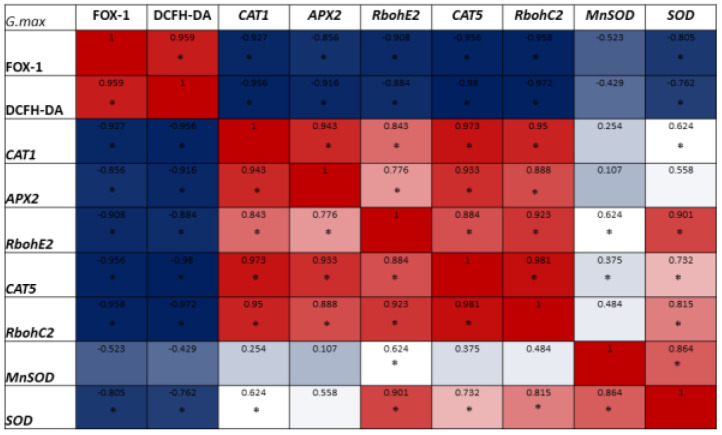
Pearson correlation indices calculated for *G. max* hydroprimed seeds taking into consideration the biochemical assays for ROS quantification (FOX-1 and DCFH-DA) and the ROS homeostasis gene (*CAT*1, *APX*2, *CAT*5, *MnSOD*, *SOD*1, *RbohE*2, *RbohC*2) expression levels. The blue color indicates negative correlations whereas red indicates positive correlations. Statistically significant correlations (*p* < 0.05) are indicated by an asterisk (*).

Subsequently, correlation analyses were performed between data obtained from the ROS detection methods (DCFH-DA and FOX-1) and gene expression profiles (Figure 6). In general, a negative correlation is observed between the gene expression and ROS levels. For example, this is observed between the data obtained in the FOX-1 analysis and the *CAT*1. *RbohE*2, *APX*2, and *SOD*1 expression levels. The same trend is observed for the data obtained with the DCFH-DA analysis and the *CAT*1, *CAT*5, *RbohE*2, *APX*2, and *SOD*1 relative expression. In contrast, positive correlations are generally observed between the gene expression data, namely for *CAT*1, *CAT*5, and *APX*2 compared to *SOD*1. Furthermore, it is interesting to note that the *RbohE*2 (gene responsible for the production of ROS in seed) expression levels also showed a positive correlation with the expression levels of all the genes responsible for ROS removal (*MnSOD*, *SOD*1, *CAT*1, *CAT*5, *APX*2). A similar trend is observed also for *RbohC*2. Finally, significantly positive correlations are observed between the ROS levels measured through the FOX-1 assay (in terms of peroxide species concentration) and the DCFH-DA (in terms of oxidative stress levels) assay.

## 4. Discussion

Currently, standard germination tests approved by ISTA represent the main methods that allow the observation of seed behavior in the postsowing phase [1,64]. The most common methods for seed quality establishment are invasive and do not allow the continued evaluation of seeds over time. Among the invasive techniques, aside from germination tests, the evaluation of moister content [65,66], tetrazolium test [67,68], and accelerated aging systems [69,70] are also used often. Most of these chemical and physical techniques exhibit a good accuracy and reliability but also present certain limitations, such as high cost, health hazards, lengthy duration, and high operator requirements [2]. These methods also raise problems related to the direct use of seeds and the time required to obtain relevant information. The development of new techniques and procedures by which to analyze seed characteristics is driven by the need to overcome these drawbacks. Therefore, several nondestructive methodologies have been developed, many of them being based on the use of different imaging techniques supported by computer vision to rapidly collect and interpret high-resolution images [3,71,72,73]. These include thermal imaging [74,75], X-rays [76,77,78], and spectroscopic techniques such as near-infrared spectroscopy (NIRS) technologies [5,79,80,81], Raman spectroscopy [3,82,83], or hyperspectral imaging [83,84]. Although these noninvasive methods represent a faster, deeper, and more precise way to retrieve important information for the evaluation of seed quality, the associated costs, and the required expertise are still prohibitive for large-scale screening of seed lots. Therefore, there is still the need to expand the palette of methods by which to reduce their costs or to promote the development of other cost-effective and sustainable methods.

Given these premises, the current study proposes two biochemical assays that can be employed to detect the levels of ROS as a proxy of seed quality. Why focus on ROS? Because, as already indicated, these are essential molecules with well-proven roles in seed dormancy and germination [5,6,7,23,24], relevant processes in the context of seed vigor and seed quality assessment. To prove that the proposed assays can be adopted as methods to test seed quality, we have first developed appropriate materials by applying treatments meant to boost (hydropriming) or inhibit (heat-shock) seed germination. We have adopted soybean as a reference species in this study because of its high agroeconomic relevance as well as possible model legume and availability of database resources [59,60]. However, to show that these approaches can be universally applied, we have extended the study to other relevant crops like tomato and wheat, hence covering seed morphological diversity. Indeed, our results show that hydropriming improved germination performance but this is conditioned by the soaking time. On the other hand, the HS treatments imposed in this study suppressed seed germination.

Having defined the experimental systems, the following step consisted of evaluating ROS levels and comparing the two proposed approaches. Interestingly, even if the DCFH-DA and FOX-1 assays relatively measure different components, namely oxidative status and H_2_O_2_ released radicals respectively, in the case of soybean the results obtained follow the same pattern: higher levels of ROS in HS and CTRL and low levels during the HP treatments. For the FOX-1 assay, this trend is also maintained in the other investigated species, whereas the DCFH-DA results were much more variable. This may be due to the different types of measurement techniques; on the one hand, the use of a fluorimeter with extracting the baseline fluorescence levels, and, on the other hand, the use of spectrophotometric readings plotted to a standard curve. Moreover, the DCFH-DA assay is generally used to quantify intracellular ROS levels [85,86,87], whereas FOX-1 is used for measuring the release of specific ROS in the surrounding environment [52,88,89]. The DCFH is usually oxidized to the fluorescent product DCF by multiple reactive species, and thus it is not specific for a particular ROS [90,91]. In addition, other limitations include the fact that DCFH is not oxidized directly by H_2_O_2_, but only after its conversion to more reactive species, and this oxidation is also sensitive to O_2_ levels, light, and pH. Consequently, several studies have indicated that the observed fluorescence may not be proportional with the accumulation of ROS [92,93]. By contrast, FOX-1 is generally used in an acidic environment, and it relies on the oxidation of Fe^2+^ to Fe^3+^ [94]. In this case, hydroperoxides oxidize the ferrous ion to ferric ion, subsequently treated with the XO reagent to generate a ferric-XO complex, resulting in a blue–purple color readable at 550–560 nm [95]. This approach has received much attention not only because of its low cost but also because it is not affected by environmental conditions (e.g., O_2_, light) [96].

To show that the applied methods are noninvasive, we have monitored the germination percentage of seeds imbibed in the DCFH-DA and FOX-1 reagents for 15 and 30 min, respectively, and no significative differences were observed between CTRL and imbibed seeds (Figure S7).

Lastly, to prove that the ROS turnover is influenced within the proposed system, a qRT-PCR approach was adopted to monitor the expression of genes encoding enzymes involved in both ROS production (*RbohE*2, *RbohC*2) and scavenging (*MnSOD*, *SOD*1, *CAT*1, *CAT*5, *APX*2). The scientific literature is rich in studies evidencing that seed priming treatments result in differential expression of a myriad of antioxidant genes [35,36,37,45,46,97,98]. And indeed, the observed upregulation of the selected genes is in agreement with the cited data. The upregulation of both ROS production and scavenging genes indicates active ROS turnover; thus while ROS are being produced the antioxidant systems are being activated. Additionally, a correlation analysis was carried out between the measured ROS through the two assays and the levels of expression of the investigated genes. The positive correlations observed between the DCHF-DA and FOX-1 data in the case of soybean seeds indicate that the different types of ROS detected by the two assays display a similar accumulation pattern. By contrast, during seed priming the expression of genes involved in ROS turnover increases while the observed levels of measured ROS decrease. This is statistically reinforced by the recurrent negative correlations observed between the ROS patterns and gene expression levels. This can be thus interpreted as an indication of the efficiency of the antioxidant response in reducing ROS accumulation.

The noninvasiveness and relative rapidity of the proposed assays can have promising outcomes in multiple experimental and applicative contexts. For instance, from an experimental point of view, these can be used to track the kinetics of ROS dynamics for individual seeds, providing a time-lapse to monitor the progression of priming protocols or the activation of the seed pre-germinative metabolism within the “oxidative window” [6]. For the applicative side, whenever seed materials are scarce these assays may allow the evaluation of the quality of a seed lot without losing valuable material. This can be applied to seed bank accessions and elite breeding materials with important implications on biodiversity preservation in crops and wild species.

## 5. Conclusions

The current study proposes two noninvasive, rapid, cost-effective, and potentially universal techniques by which to measure ROS production in seeds as a proxy of seed quality evaluation. Although the DCFH-DA assay is more variable and subjected to certain limitations in terms of types of measured ROS and interaction with surrounding factors, the FOX-1 approach appears to be more reliable when applied to different types of seeds. Additional proof of the accuracy of the investigated methods is provided through the correlation analysis performed, taking into consideration the measured ROS levels and the expression of genes involved in ROS turnover. To further validate the obtained data, the methods could be subsequently applied to other species, varieties/genotypes, seed types, and experimental conditions, such as different seed lots collected from diverse environments, seed storage conditions, seed aging protocols, and damaging or beneficial treatments.

## Figures and Tables

**Table 1:** Germination parameters calculated for *Glycine max*. Statistical differences among treatments and control are represented with asterisks (*), *p* < 0.05. Formulas and measure units for each parameter are provided in the supplementary materials. PV, peak value; MGT, mean germination time; MGR, mean germination rate; CVG coefficient of velocity; U, uncertainty index; Z, synchronicity index; CTRL, nontreated control; HP2, hydropriming for 2 h; HP4, hydropriming for 4 h; HP8, hydropriming for 8 h; HS, heat-shock.

	CTRL	HP2	HP4	HP8	HS
**PV**	6.67 ± 0.76	11.67 ± 0.58 *	14 ± 2.65 *	14 ± 2 *	0 ± 0 *
**MGT**	2.18 ± 0.09	1.41 ± 0.06 *	1.22 ± 0.12 *	1.33 ± 0.17 *	n.d.
**MGR**	0.46 ± 0.02	0.71 ± 0.03 *	0.82 ± 0.08 *	0.76 ± 0.10 *	n.d.
**CVG**	45.89 ± 1.87	70.98 ± 2.76 *	82.24 ± 7.84 *	75.7 ± 9.95 *	n.d.
**U**	1.28 ± 0.08	1.1 ± 0.13	0.72 ± 0.21 *	1 ± 0.34	0 ± 0 *
**Z**	0.43 ± 0.02	0.48 ± 0.03	0.65 ± 0.13 *	0.56 ± 0.15	0 ± 0 *

## Data Availability

The data presented in this study are available in the article and supplementary materials.

## Supplementary Materials

The following supporting information can be downloaded at: https://www.mdpi.com/article/10.3390/antiox12030626/s1. Table S1: Germination parameters formulas; Table S2: List of oligonucleotides sequences used for the qRT-PCR analysis; Table S3: Germination parameters calculated for *S. lycopersicum*, and *T. aestivum*; Figure S1: FOX-1 calibration curve; Figure S2: Representative images of *S. lycopersicum* and *T. aestivum* HP germinated seedlings. Figure S3: Evaluation of HP efficiency in tomato and wheat seeds; Figure S4: ROS detection in *S. lycopersicum* and *T. aestivum* seeds subjected to HP and HS treatments; Figure S5: PCA analysis for wheat and tomato; Figure S6: Gene expression heatmaps in seeds of *G. max* and *A. thaliana*; Figure S7: Germination percentage (G%) for soybean, tomato and wheat seeds imbibed in DCFH-DA and FOX-1.
