# Supplementary material for: Noninvasive Methods to Detect Reactive Oxygen Species as a Proxy of Seed Quality
Source: Antioxidants (Basel). 2023 Mar 3;12(3):626. doi: 10.3390/antiox12030626 (PMC10045522; doi:10.3390/antiox12030626)
Supplement: Supplementary file 1 [file antioxidants-12-00626-s001.zip › antioxidants-2241591-supplementary.pdf]

Non-Invasive Methods to Detect Reactive Oxygen Species as a Proxy of Seed Quality

Adriano Griffo[1], Nicola Bosco[1], Andrea Pagano[1], Alma Balestrazzi[1,2], and Anca Macovei[1,2*]

[1] Department of Biology and Biotechnology 'L. Spallanzani', University of Pavia, Via Ferrata 9, 27100 Pavia, Italy

[2] National Biodiversity Future Center (NBFC), Palermo 90133, Italy

*Correspondence: anca.macovei@unipv.it

Supplementary Table S1. Germination parameters used in this study to assess seed germination as reported by Ranal and Garcia de Santana (2006). For each parameter, definition, formula, limits of measurement and unit are shown.

Parameter	Formula	Limits	Unit
G<br>mean number of germinated seeds per day expressed in percentage	*G* = (100* n. of germinated seeds)/ Total n. of seeds	$0 \leq G \leq 100$	%
PV<br>Peak Value	Rate = {Ni/Ti = Nmax}	$0 \leq PV \leq N$	N/day
MGT<br>mean germination time (*)	$\bar{t} = \sum_{i=1}^{k} n_i t_i / \sum_{i=1}^{k} n_i$	$0 < t \leq k$	day
CVG<br>coefficient of velocity of germination	$CVG = (\sum_{i=1}^{k} f_i / \sum_{i=1}^{k} f_i x_i)100$	$0 < CVG \leq 100$	%
MGR<br>mean germination rate	$v = CV/100$	$0 < v \leq 1$	day^{-1}
U<br>uncertainty associated to the distribution of the relative frequency of germination (**)	$\bar{E} = -\sum_{i=1}^{k} f_i \log_2 f_i$, being $f_i = n_i / \sum_{i=1}^{k} n_i$	$0 \leq U \leq \log_2 n$	bit
Z<br>synchronization index (***)	$Z = \sum C_{n_i, 2} / N$	$0 \leq Z \leq 1$	Unit less

(*) t_i is time from the start of the experiment to the ith observation (day); n_i: number of seeds germinated in the time *i* (not the accumulated number, but the number correspondent to the ith observation), and *k* is the last time of germination.
(**) f_i is the relative frequency of germination, n_i the number of seeds germinated on the day *i*, and *k* the last day of observation
(***) $C_{ni, 2}$: combination of the seeds germinated in the time *i*, two together, and n_i the number of seeds germinated in the time *i.*

Supplementary Table S2. List of oligonucleotides sequences used for the qRT-PCR analysis.

Gene	Phytozome Accession No.	Primer Forward (5'-3')	Primer Reverse(5'-3')
MnSOD	Glyma.04G221300	GGTGTGGCTTGGTCTGG	CATGCTCCCAAACATCAATACC
SOD1	Glyma.19G240400	GAGGGTGTCACTGGAACTATTTTC	GTAGTGTCCCCCAAGGCA
CAT5	Glyma.06G017900	GAACGTGTTGTCCATGCCA	GCTACCACGCTCATGAATGAC
CAT1	Glyma.17G261700	CTCATCGTCCGTTTCTCCA	GTGGGACTTGGGGTTGG
APX2	Glyma.12G073100	CCGTTGAGAAGGCGAAGAAG	CGGAGGGGTGCTTTATGG
RbohE2	Glyma.08G005900	GAGGGCAAGAGAGGGTGAG	AAGAGCAGAGCGAGCATCA
RbohC2	Glyma.06G162300	TTTCTATAACCTCCGCCCCT	GTCCACTCTTGCCGTTGTC
CYP	Glyma.12G024700	ACGACGAAGACGGAGTGG	CGACGACGACAGGCTTGG
RP40S	Glyma05G37470	TTCCACCTCGCAACCATGAT	CGAAGCAAACTCCCTCTTGG

Supplementary Table S3. Germination parameters calculated for *Solanum lycopersicum*, and *Triticum aestivum*. Statistical differences among treatments and control are represented with asterisks (*), $p < 0.05$. PV, peak value; MGT, mean germination time; MGR, mean germination rate; CVG coefficient of velocity; U, uncertainty index; Z, synchronicity index; CTRL, non-treated control; HP2, hydro-priming for 2 h; HP4, hydropriming for 4 h; HP8, hydropriming for 8 h; HS, heat shock.

S.lycopersicum	CTRL			HP2			HP8			HP24		
	Media		St. dev.	Media		St. dev	Media		St. dev	Media		St. dev.
Peak Value	6,64	±	0,97	8,56	±	0,50*	7,89	±	0,96	8,00	±	0,88
MGT	3,35	±	0,05	3,08	±	0,12*	3,11	±	0,07*	3,18	±	0,14
MGR	0,30	±	0,00	0,33	±	0,01*	0,32	±	0,01*	0,31	±	0,01
CVG	29,85	±	0,43	32,50	±	1,28*	32,17	±	0,67*	31,46	±	1,38
U	0,98	±	0,13	0,76	±	0,21	0,90	±	0,20	0,98	±	0,14
Z	0,56	±	0,03	0,73	±	0,08*	0,66	±	0,08	0,66	±	0,05*

T.aestivum	CTRL			HP2			HP4			HP6		
	Media		St. dev.	Media		St. dev	Media		St. dev	Media		St. dev.
Peak Value	7,00	±	1,80	6,06	±	1,23	6,00	±	1,80	6,17	±	0,76
MGT	2,07	±	0,11	1,99	±	0,48	1,94	±	0,30	1,91	±	0,21
MGR	0,48	±	0,03	0,52	±	0,13	0,52	±	0,08	0,53	±	0,06
CVG	48,41	±	2,56	52,40	±	13,23	52,29	±	8,15	52,68	±	5,53
U	1,28	±	0,44	1,44	±	0,40	1,58	±	0,19	1,54	±	0,22
Z	0,50	±	0,18	0,36	±	0,09	0,31	±	0,06	0,33	±	0,06

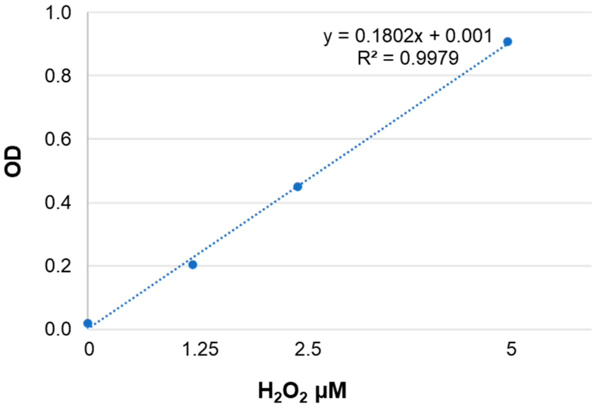

Supplementary Figure S1. FOX-1 calibration curve performed with several concentrations (0, 1.25, 2.50, 5 µM) of hydrogen peroxide (H_2O_2).

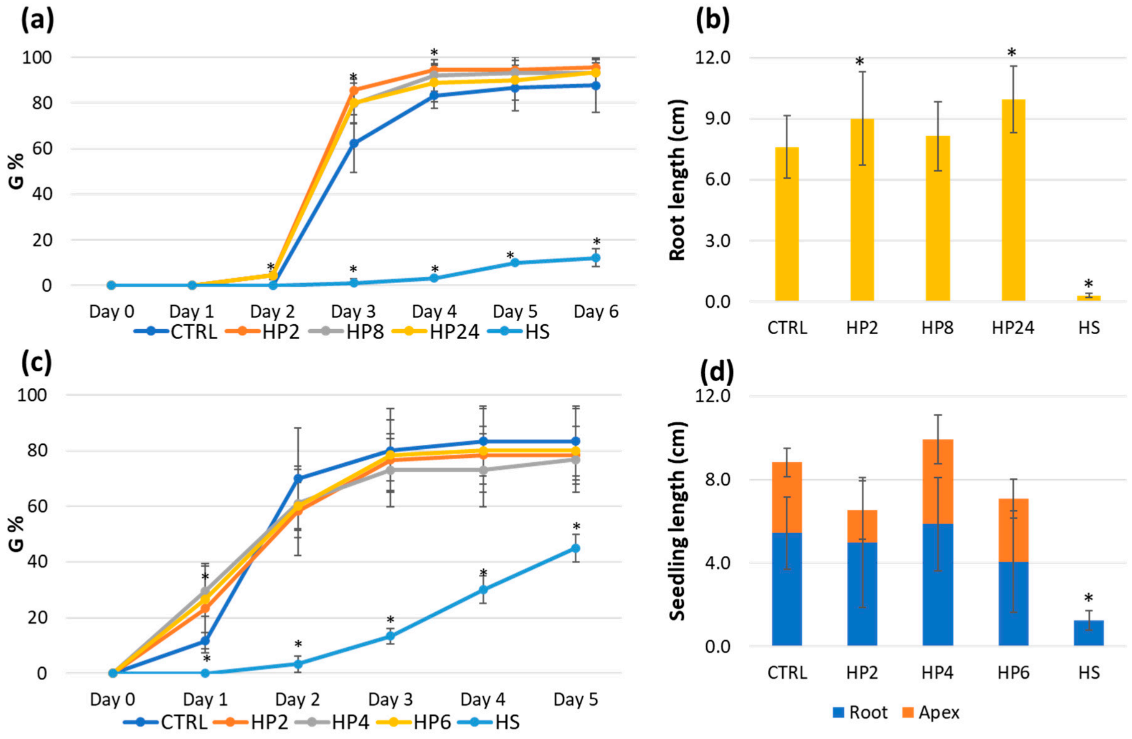

Supplementary Figure S2. Evaluation of hydropriming efficiency in tomato (*Solanum lycopersicum*) and wheat (*Triticum aestivum*) seeds. **(a)** Germination percentage (%) measured daily in tomato for a time period of six days. **(b)** Root length (cm) measured in 6-days-old tomato seedlings. **(c)** Germination percentage (%) measured daily in wheat for a time period of five days. (d) Seedling length (cm) measured terms of roots and aerial parts in 5-days-old wheat plantlets. Statistical differences among treatments and control are represented with asterisks (*), $p < 0.05$. CTRL, non-treated control; HP2, hydropriming for 2 h; HP4, hydropriming for 4 h; HP6, hydropriming for 6 h; HP8, hydropriming for 8 h; HP24, hydropriming for 24 h; HS, heat shock.

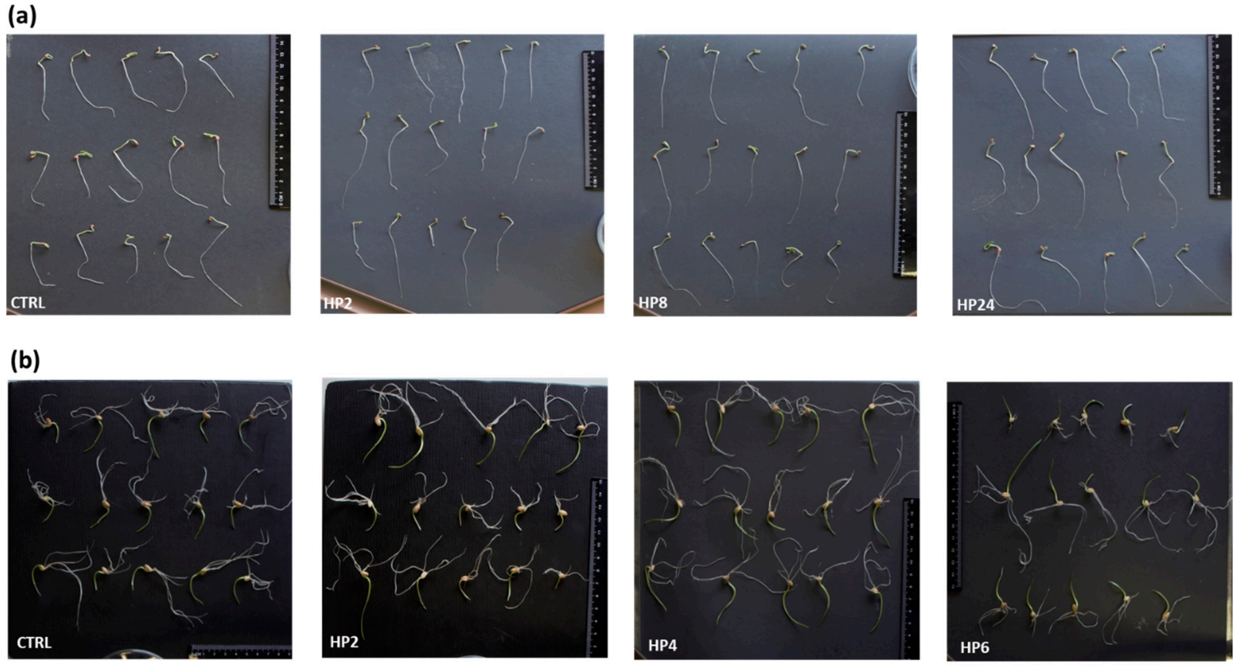

Supplementary Figure S3. **(a)** Representative images of germinated *S. lycopersicum* seedlings taken at the sixth day after hydropriming treatments. (b) Representative images of germinated *T. aestivum* seedlings taken at the fifth day after hydropriming treatments. CTRL, non-treated control; HP2, hydropriming for 2 h; HP4, hydropriming for 4 h; HP6, hydropriming for 6 h; HP8, hydropriming for 8 h; HP24, hydropriming for 24 h; HS, heat-shock.

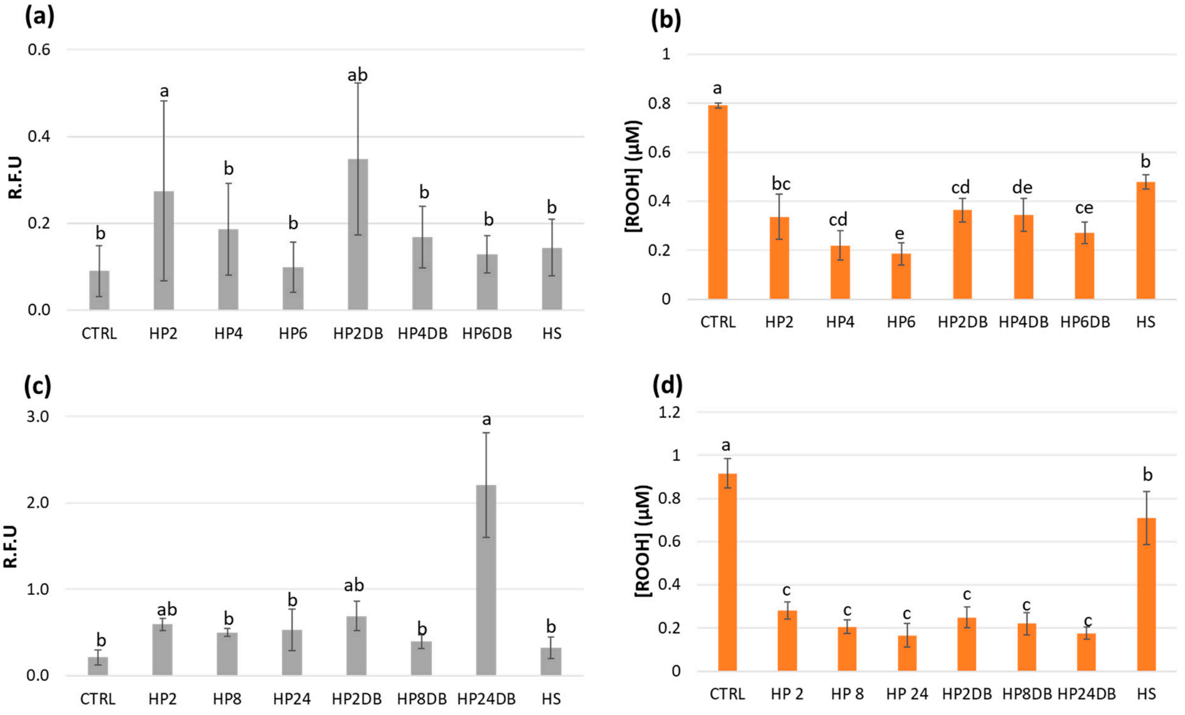

Supplementary Figure S4. ROS detection in *Solanum lycopersicum* and *Triticum aestivum* seeds subjected to hydropriming and heat shock treatments. **(a)** Levels of relative fluorescence units (RFU) measured with the DCFH-DA assay in *T. aestivum*. **(b)** ROS levels as revealed by FOX-1 assay in *T. aestivum*. Data are reported as concentrations of hydrogen peroxide and peroxyl radicals [ROOH] in µM. **(c)** Levels of relative fluorescence units (RFU) measured with the DCFH-DA assay in *S. lycopersicum*. **(d)** ROS levels as revealed by FOX-1 assay in *S. lycopersicum*. Statistically significant differences ($p < 0.05$) are indicated by the occurrence of different letters. CTRL, non-treated control; HP2, hydropriming imbibition for 2 h; HP4, hydropriming imbibition for 4 h; HP8, hydropriming imbibition for 8 h; HP-DB, dry-back treatment following hydropriming imbibition; HS, heat shock.

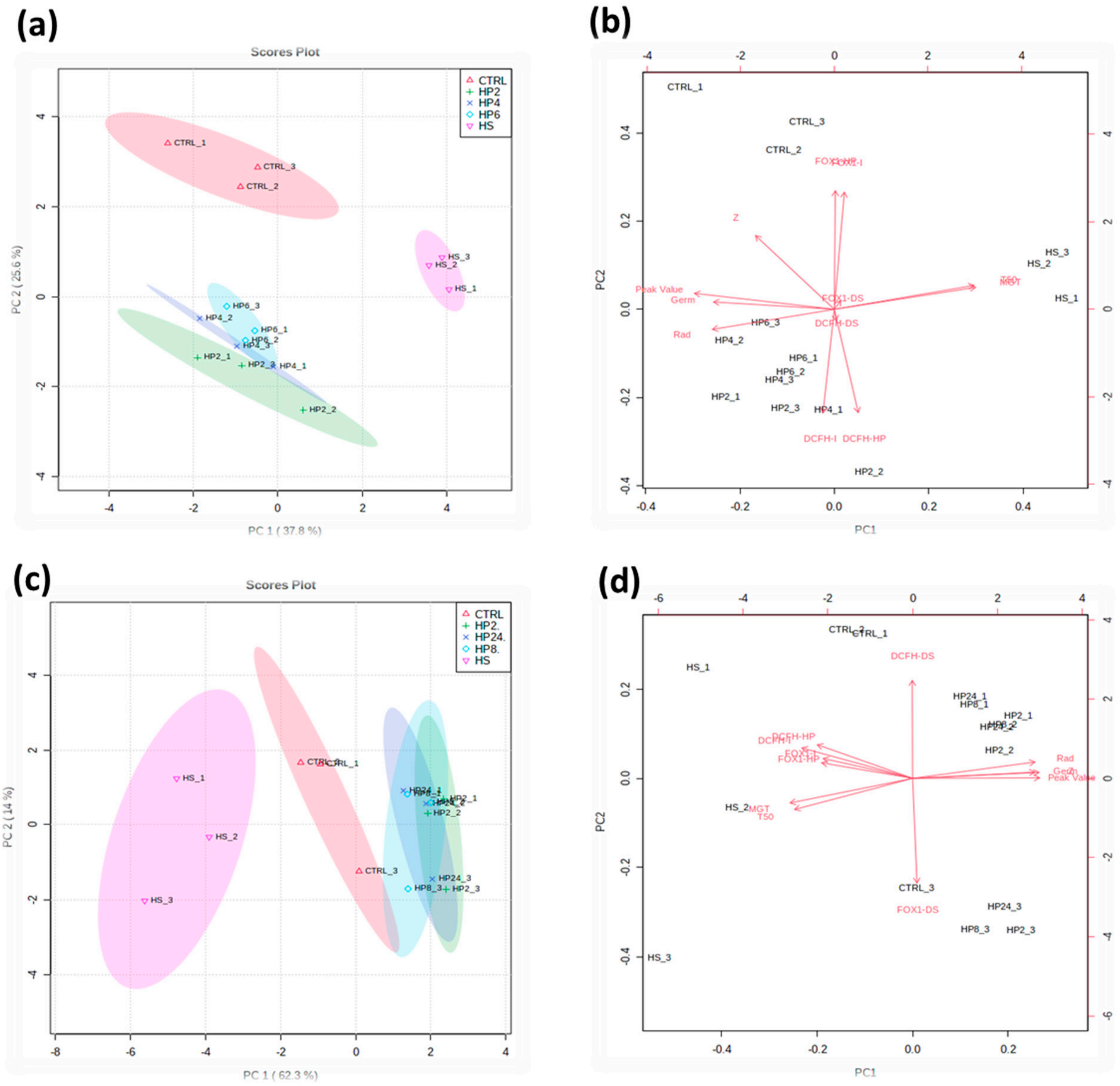

Supplementary Figure S5. Principal Component Analysis using data gathered for the imposed treatments (CTRL, HP2, HP4, HP8, HS) for *Triticum aestivum* and *Solanum lycopersicum*. (a) Scores plot grouping of samples subjected to different treatments for *T. aestivum*. (b) Bi-plot incidence of data obtained from germination tests (G, PV, MGT, Z, Rad) and ROS measurements (FOX-1, DCHF-DA) on the clustering of the groups subjected to the different treatments *T. aestivum*. (c) Scores plot grouping of samples subjected to different treatments for *S. lycopersicum*. (d) Bi-plot incidence of data obtained from germination tests (G, PV, MGT, Z, Rad) and ROS measurements (FOX-1, DCHF-DA) on the clustering of the groups subjected to the different treatments *S. lycopersicum.*

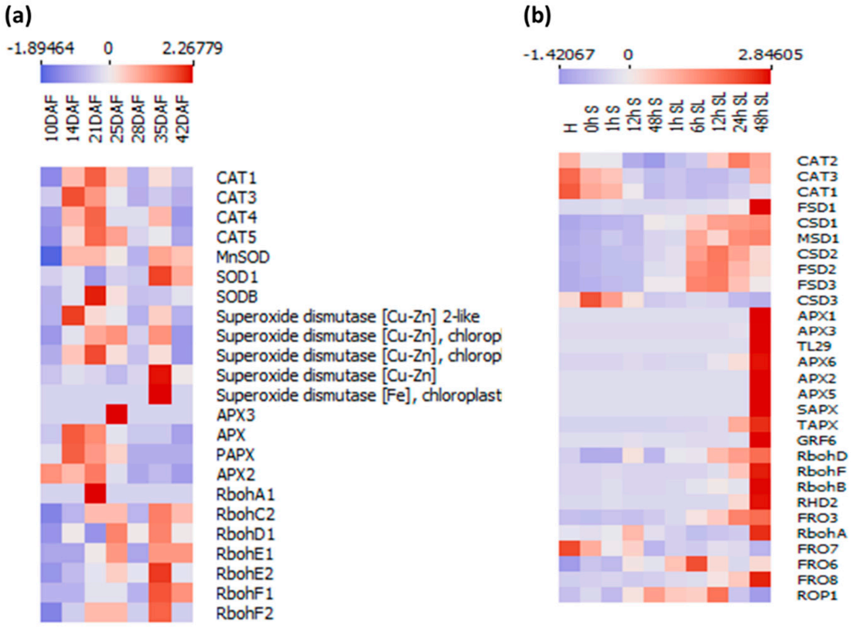

Supplementary Figure S6. Heatmaps evidencing relative expression patters of multiple homologue genes involved in ROS scavenging (*SOD, CAT, APX*) and production (*Rboh*) in seeds of *G. max* **(a)** and *A. thaliana* **(b)**. Expression values were collected from the BAR ePLANT database (http://bar.utoronto.ca/eplant) and used after Z score transformation to generate heatmaps using the Orange (https://orangedatamining.com/) online tool. Blue color indicates gene downregulation whereas red color indicates gene upregulation.

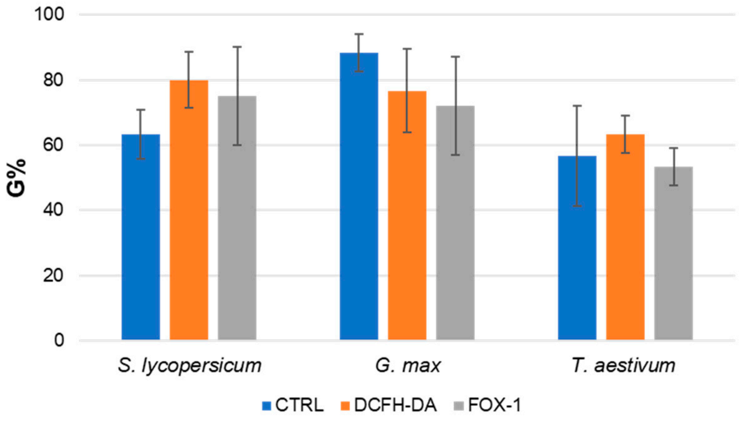

Supplementary Figure S7. Germination percentage (G%) evaluated for *S. lycopersicum*, *G. max*, and *T. aestivum* seeds imbibed in the presence of DCFH-DA and FOX-1 for 15 min and 30 min, respectively. The number of germinated seed was monitored for three days and the data collected at the 3rd day was used to generate the graphic. Statistical differences among treatments and control are represented with asterisks (*), $p < 0.05$.
